# The Involvement of HIF-1α and BDNF in Neonatal Hypoxic–Ischemic Insult to the Cerebral Germinal Matrix

**DOI:** 10.3390/ijms27115125

**Published:** 2026-06-05

**Authors:** Felipe Paes Gomes da Silva, Francys De Luca Fernandes da Silva, Nicolas Pereira Gerber, Seigo Nagashima, Eduardo Morais de Castro, Vanessa Yumie Watanabe Liberalesso, Carlos Frederico Oldenburg Neto, Gustavo David dos Santos, Fernanda Gimenez de Souza, Ana Paula Camargo Martins, Lucia de Noronha, Caroline Busatta Vaz de Paula

**Affiliations:** 1 Laboratory of Experimental Pathology, Pontifícia Universidade Católica do Paraná (PUCPR), Curitiba 80215-901, Brazil; paes.silva@pucpr.edu.br (F.P.G.d.S.); francys.silva@pucpr.edu.br (F.D.L.F.d.S.); nicolas.gerber@pucpr.edu.br (N.P.G.); seigo.nagashima@pucpr.br (S.N.); eduardo.castro@ufpr.br (E.M.d.C.); vanessa.salomao@pucpr.br (V.Y.W.L.); frederico.oldenburg@pucpr.br (C.F.O.N.); g.david@pucpr.br (G.D.d.S.); f.gimenez@pucpr.br (F.G.d.S.); ana.martins@pucpr.br (A.P.C.M.); 2 Postgraduate Program of Health Sciences, School of Medicine and Life Sciences, Pontifícia Universidade Católica do Paraná (PUCPR), Curitiba 80215-901, Brazil; 3 Department of Medical Pathology, Universidade Federal do Paraná (UFPR), Curitiba 80060-900, Brazil

**Keywords:** neonatal hypoxia, germinal matrix, BDNF, HIF-1α, preterm birth, neuroprotection, asphyxia, neuronal injury, biomarker, hypoxic-ischemic encephalopathy

## Abstract

Perinatal asphyxia is a major contributor to neonatal morbidity and mortality, particularly among preterm infants, whose brains are highly vulnerable to hypoxic–ischemic injury. The germinal matrix (GM), owing to its vascular fragility and high metabolic demand, is especially susceptible in this context. This study analyzed 118 germinal matrix samples from neonates, stratified into three groups according to gestational age—Extremely Preterm (EP), Late Preterm (LP), and Term (T)—to investigate the immunopositivity of hypoxia-inducible factor 1-alpha (HIF-1α) and brain-derived neurotrophic factor (BDNF), correlating these findings with gestational age, the presence of asphyxia, neuronal injury, and survival time. BDNF expression showed a positive association with postnatal survival in neonates without neuronal injury (ρ = 0.309; *p* = 0.012). Linear regression analysis further demonstrated that BDNF immunopositivity was a significant predictor of survival time, with each 11.82% increase in positive staining corresponding to an additional predicted hour of survival (*p* < 0.001). HIF-1α expression was positively associated with survival in asphyxiated extremely preterm neonates (ρ = 0.492; *p* = 0.024) and demonstrated a strong correlation that approached, but did not reach, conventional statistical significance in late preterm neonates with neuronal injury (ρ = 0.949; *p* = 0.051). Collectively, these findings suggest a complementary role for BDNF and HIF-1α in neonatal neuroprotective responses, with BDNF showing potential as a prognostic biomarker in neonates without neuronal injury and HIF-1α reflecting adaptive responses to hypoxic–ischemic stress in a gestational age-dependent manner. However, additional studies are required to validate these associations and further clarify their prognostic and therapeutic relevance in neonatal hypoxic–ischemic conditions.

## 1. Introduction

Infant mortality is recognized as an important indicator of a population’s socioeconomic, sanitary, and environmental conditions [1]. According to the 2023 UNICEF report released by the United Nations Inter-Agency Group for Child Mortality Estimation, approximately 4.9 million children died before reaching five years of age in 2022—a reduction of one hundred thousand compared to 2021 figures [2,3]. In Brazil, this rate was approximately 10.6 deaths per 1000 live births in 2021, according to the latest published epidemiological bulletin, which underscores the issue as a public health priority [4,5].

Neonatal death, one of the main components of infant mortality, is defined as the death of a newborn within the first 28 days of life—a particularly critical period for child survival, accounting for 47% of all deaths among children under five years old [6]. In this context, prematurity, low birth weight, and perinatal asphyxia are among the leading causes of neonatal mortality [7].

Among these conditions, perinatal asphyxia (PA) stands out not only as the third-leading cause of early neonatal mortality but also as a syndrome resulting from a hypoxic episode that may occur before, during, or shortly after delivery [8,9]. When prolonged, oxygen deprivation impairs the body’s ability to maintain adequate systemic perfusion, potentially leading to multiorgan dysfunction and, ultimately, neonatal death [10].

Within this framework, the Apgar score—endorsed and iteratively refined by the American Academy of Pediatrics (AAP) and the American College of Obstetricians and Gynecologists (ACOG) [11]—constitutes a pivotal, standardized instrument for the rapid assessment of neonatal clinical status at the one- and five-minute postnatal intervals [12]. By quantifying five distinct physiological parameters—skin color, heart rate, reflex irritability, muscle tone, and respiratory effort—the score facilitates an immediate appraisal of cardiovascular, pulmonary, and neurological integrity [13,14]. Given its multidimensional nature, the Apgar test serves as a critical clinical indicator of perinatal hypoxic–ischemic insults which, despite their primary respiratory manifestation, frequently precipitate deleterious multisystemic consequences [15,16,17,18,19,20].

Neurological sequelae represent the most clinically profound ramifications of such events. While an isolated Apgar score does not provide a definitive diagnosis of asphyxia, a score of 3 or less at the five-minute mark is widely recognized as a robust clinical harbinger of heightened risk for neonatal encephalopathy [11,15]. In such instances, prolonged oxygen deprivation triggers a cascade of biochemical perturbations in neuronal function, culminating in metabolic acidosis and the subsequent pathophysiological progression of hypoxic–ischemic encephalopathy. This susceptibility to hypoxic–ischemic injury is markedly exacerbated in preterm neonates, whose neurological immaturity and impaired cerebral autoregulation significantly increase the vulnerability of transient structures, such as the germinal matrix (GM), to hypoxic insults [10,17,18,19].

The germinal matrix is a transient structure during embryonic development [21], located primarily in the periventricular region of the lateral ventricles. It consists of subependymal cells that, between the 10th and 20th weeks of gestation, serve as an important mitotic center for neuronal precursors such as neuroblasts and glioblasts—the latter being mainly active in the second half of gestation [12,13].

The involution of the germinal matrix begins progressively around the 24th week of gestation and regresses significantly by approximately the 36th week. Notably, although the GM is highly vascularized, its histological architecture is fragile, characterized by small-caliber vessels, immature endothelial junctions, and basal lamina, absence of a muscular layer, and lack of direct contact with perivascular glial structures—factors that contribute to its high vulnerability under oxygen-deprived conditions [13,14,20].

Despite this immaturity, the GM compensates through vigorous angiogenesis, serving as an adaptive response to the region’s high metabolic demand. This angiogenesis is supported not only by dense vascularization but also by a high rate of endothelial turnover [13,19]. This characteristic is driven by elevated concentrations of Vascular Endothelial Growth Factor (VEGF), a signaling protein that is significantly more abundant in the GM than in the cerebral cortex or white matter. In GM, VEGF plays a fundamental role in the migration, proliferation, and increased permeability of endothelial cells that make up the vascular structure in this region [22].

One of the key regulatory mechanisms of VEGF-mediated angiogenesis is governed by oxygen-sensitive intracellular pathways, notably involving the transcription factor hypoxia-inducible factor 1-alpha (HIF-1α) [22]. HIF-1α is a protein that dimerizes with HIF-1β, and its stability is closely linked to oxygen levels. Under normoxic conditions, specific proline residues in HIF-1α are hydroxylated, facilitating its recognition by the von Hippel–Lindau protein (pVHL), which tags it for ubiquitination and subsequent degradation via the ubiquitin–proteasome pathway [23]. Conversely, under hypoxic conditions, HIF-1α subunits accumulate in the nucleus, where they bind to ARNT (Aryl Hydrocarbon Receptor Nuclear Translocator)—a protein constitutively expressed regardless of oxygen levels—activating the transcription of numerous genes involved in cellular adaptation [23].

Ultimately, HIF-1α plays a crucial role under hypoxic stress, enabling cells to adapt through the upregulation of genes involved in mitochondrial autophagy (to reduce reactive oxygen species), erythropoiesis (to enhance oxygen transport), and angiogenesis [23,24,25,26].

Moreover, emerging evidence suggests an additional role for HIF-1α in modulating the expression of neurotrophic factors, which are essential for neurodevelopment and neuroprotection in neonates experiencing hypoxic–ischemic events. Among these, the brain-derived neurotrophic factor (BDNF) stands out. BDNF belongs to the family of neurotrophins and is initially synthesized in the endoplasmic reticulum as a precursor, pro-BDNF [27,28]. After synthesis, the molecule is transported to the Golgi apparatus and secreted by postsynaptic dendrites in response to neuronal activity. Cleavage of the terminal domain of pro-BDNF yields the mature, biologically active form—mBDNF [28,29]. Functionally, BDNF promotes not only neuronal development—supporting neuroblast survival and migration along glial precursors during the survival and growth phases—but also synaptic plasticity [27,30].

Given the evidence regarding the roles of HIF-1α and BDNF in neural development and the response to cerebral injury, it becomes essential to investigate how these pathways influence cell differentiation and survival in the germinal matrix following hypoxic–ischemic events. This study aims to investigate the molecular responses associated with hypoxic–ischemic stress in the germinal matrix, focusing on the expression of HIF-1α and BDNF as potential markers of adaptive cellular responses within the pathological continuum that may precede neuronal injury or vascular disruption.

## 2. Results

### 2.1. Clinical Profile of the Samples

The study analyzed 118 neonatal autopsy cases. Detailed demographic data and clinical characteristics—including sex distribution, gestational age groups, the prevalence of perinatal asphyxia, and postnatal survival time—are summarized in Table 1. Notably, 28.8% (n = 34) of the neonates met the clinical criteria for perinatal asphyxia (Apgar score ≤ 3 at 5 min). Regarding longevity, the majority of the sample survived between 25 and 72 h (40.7%), with a total mean survival time of 4.4 ± 8 h. The primary neuropathological findings observed across the sample included periventricular hemorrhage and cerebral edema, as detailed in the anatomopathological reports (Table 1).

### 2.2. BDNF Immunopositivity

**Figure 1:**
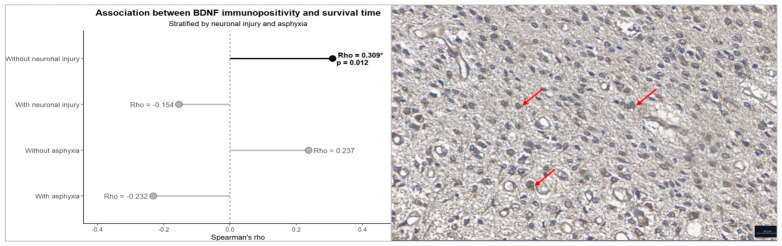
Correlation between BDNF immunopositivity and postnatal survival time in the germinal matrix. Spearman’s rho (ρ) values are shown according to neuronal injury and perinatal asphyxia status. A significant positive correlation was observed in neonates without neuronal injury (ρ = 0.309, *p* = 0.012), while no significant correlations were found in the other subgroups. The dashed vertical line indicates no correlation (ρ = 0), and the asterisk (*) denotes statistical significance. The black marker and line indicate the statistically significant association, gray markers and lines indicate non-significant associations, and red arrows indicate BDNF positive immunostaining.

A comparative analysis was performed to investigate the association between tissue BDNF immunoexpression and clinical outcomes. When neonates from the Extremely Preterm Group (EP), Late Preterm Group (LP), and Term Group (T) were analyzed together, the correlation between tissue BDNF immunoexpression and survival time (hours) was evaluated in subgroups defined by the presence or absence of asphyxia and neuronal injury; a significant positive correlation was observed between BDNF immunoexpression and survival time (hours) in neonates without neuronal injury (ρ = 0.309; *p* = 0.012; Figure 1). No statistically significant correlations were observed in the remaining subgroups defined by the presence or absence of asphyxia and neuronal injury.

To further explore this relationship, a linear regression model was applied. The analysis revealed that BDNF immunopositivity was a significant predictor of longevity; for every 11.82% increase in positive staining, an additional hour of survival could be predicted (*p* < 0.001). This establishes BDNF as an independent variable associated with postnatal survival in this clinical context (Table 2).

### 2.3. HIF-1α Immunopositivity

**Figure 2:**
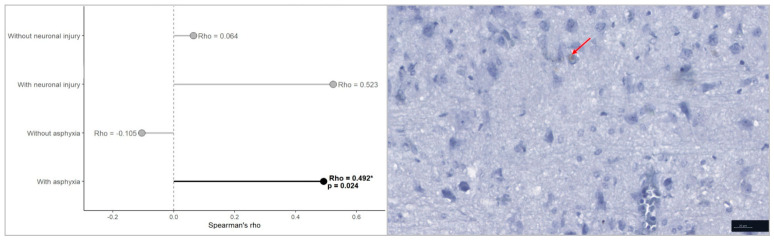
Correlation between HIF-1α immunopositivity and postnatal survival time in extremely preterm neonates. Spearman’s correlation coefficients Rho (ρ) are shown according to neuronal injury and perinatal asphyxia status. A significant positive correlation between HIF-1α immunopositivity and survival time was observed in asphyxiated neonates (ρ = 0.492, *p* = 0.024). No significant correlations were identified in the other subgroups. The dashed vertical line indicates no correlation (ρ = 0), and the asterisk (*) denotes statistical significance. The black marker and line indicate the statistically significant association, gray markers and lines indicate non-significant associations, and red arrows indicate HIF-1α positive immunostaining.

A comparative analysis was conducted to assess the relationship between HIF-1*α* immunopositivity and survival time across different clinical strata. In neonates categorized as asphyxiated, a significant positive correlation was identified between HIF-1*α* levels and survival time within the EP group (ρ = 0.491; *p* = 0.024; Figure 2).

**Figure 3:**
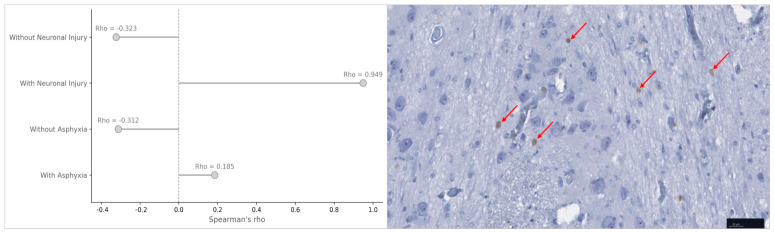
Correlation between HIF-1α immunopositivity and survival time in the late preterm group. Spearman’s correlation analysis between HIF-1α-positive staining area (%) and postnatal survival time (hours) in the late preterm cohort (LP; 31–36 weeks), stratified according to neuronal injury and perinatal asphyxia status. A strong positive correlation was observed in the subgroup with neuronal injury (ρ = 0.949), showing a trend toward statistical significance (*p* = 0.051). Representative photomicrograph of HIF-1α immunoreactivity in the germinal matrix is shown on the right, demonstrating cytoplasmic HIF-1α immunostaining in a neonate from the asphyxia subgroup. Red arrows indicate HIF-1α-positive cells.

In the LP group, a strong positive correlation was observed between HIF-1α levels and survival time in neonates with confirmed neuronal injury (ρ = 0.949; Figure 3). Despite the magnitude of the correlation coefficient, the *p*-value (0.051) was slightly above the conventional threshold for statistical significance. In light of the small sample size of this subgroup, this finding should be interpreted with caution. No similar correlations were observed in the remaining subgroups.

### 2.4. Integration of Biomarkers and Brain Maturity

**Figure 4:**
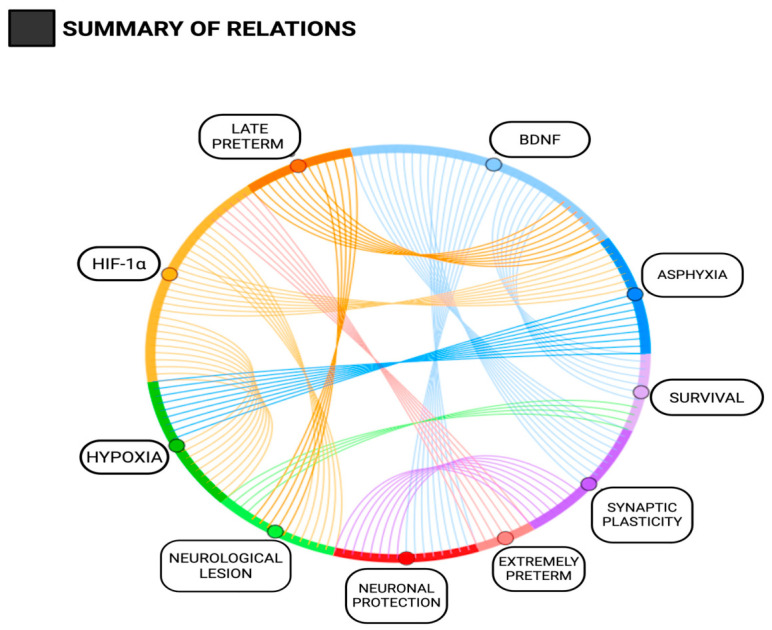
Chord diagram illustrating the multifaceted interactions between cerebral maturity, hypoxic–ischemic stress, and biological markers. The arcs represent significant statistical relationships identified within the EP and LP subgroups in relation to clinic-pathological variables, including perinatal asphyxia, survival time, and neuronal injury. The thickness and trajectory of the chords reflect the integration of BDNF and HIF-1α immunopositivity within neuroprotective and synaptic plasticity pathways. This visual synthesis highlights the distinct metabolic response patterns in the germinal matrix of preterm neonates. Quantitative details of these interactions are provided in the supplementary data.

To visualize the complex interactions between gestational maturity, hypoxic–ischemic insults, and the biological markers investigated, a chord diagram was constructed (Figure 4). This integrative model illustrates statistically significant connections revealing direct and indirect influences on neuroprotection, synaptic plasticity, and survival across the three primary axes: Extremely Preterm, Late Preterm, and Term neonates.

The thickness and trajectory of the chords reflect the strength of the correlations between clinical variables and immunopositivity levels. Notably, the diagram highlights distinct patterns of germinal matrix response according to cerebral maturity, with more robust and complex interactions observed in the preterm subgroups (EP and LP) compared with term neonates. This visual synthesis supports the hypothesis of a maturity-dependent metabolic and protective response to perinatal stress.

The chord diagram provides an integrative visualization of statistically significant associations identified in the quantitative analyses and does not constitute an independent statistical test. Quantitative details of these interactions are provided in the supplementary data.

## 3. Discussion

### 3.1. Brain Maturity Profile and Effects on BDNF and HIF-1α Expression

Fetal development is characterized by gestational age milestones, each responsible for the formation of specific organs. While these milestones are well-documented in the literature, the understanding of organ-specific developmental phases—particularly the brain—remains insufficient, especially regarding molecular-level processes at each gestational stage [31]. To address this gap, we stratified our study into gestational age subgroups to analyze brain development chronologically (Figure 4).

EP neonates exhibit a high degree of central nervous system (CNS) immaturity, particularly within the germinal matrix, rendering them susceptible to hypoxic or infectious injuries [31]. In contrast, late preterm infants, although still classified as preterm, display relatively greater brain maturity and physiological resilience, while term infants possess a more developed CNS, though they remain vulnerable to adverse perinatal conditions such as perinatal asphyxia [32,33].

Although developmental changes within the germinal matrix occur continuously across gestational weeks—particularly with respect to progenitor proliferation and neuronal migration—our grouping strategy followed clinically established categories of prematurity. This approach allowed the investigation of molecular responses across major developmental stages while preserving statistical robustness.

Importantly, the germinal matrix should not be conceptualized as equivalent to mature white matter or selectively neuronal regions such as the hippocampus. Although anatomically contiguous with the periventricular white matter, the germinal matrix represents a transient developmental compartment composed predominantly of neuronal and glial progenitor cells within a highly vascularized and structurally immature microenvironment. Its microvascular architecture is characterized by thin-walled vessels, limited perivascular glial support, and ongoing angiogenic activity, distinguishing it fundamentally from the stabilized vascular networks of mature white matter and differentiated neuronal structures. Consequently, the molecular patterns observed in the present study should be interpreted within the specific biological context of the germinal matrix, rather than extrapolated to fully differentiated cortical or hippocampal tissue [19,21].

Our findings revealed that LP group without asphyxia or injury had higher BDNF levels compared to EP (*p* = 0.025) and T (*p* = 0.046), suggesting that greater brain maturity—coupled with the absence of asphyxia—favors BDNF production and preservation [32,34]. Conversely, EP infants exposed to insults such as perinatal asphyxia showed reduced BDNF expression, likely due to the heightened fragility of the immature CNS under hypoxic stress [33,35]. These results reinforce the concept that greater brain immaturity correlates with increased vulnerability, though neuroprotective mechanisms (e.g., BDNF modulation) may be stimulated within specific developmental windows.

For HIF-1α, no statistically significant differences were observed among EP, LP, and T groups. However, positive correlations were suggested in specific subgroups, indicating that asphyxiated EP and injured LP infants may activate HIF-1α more robustly in critical scenarios [36]. This aligns with the literature highlighting the adaptive capacity of the preterm CNS to mobilize molecular survival pathways—modulating both neurotrophic factors (e.g., BDNF) and HIF-1α-associated cascades—in response to hypoxic insults [37]. Thus, the degree of prematurity emerges as a key determinant of neonatal compensatory responses, whether via BDNF production or HIF-1α activation.

Collectively, these findings underscore BDNF’s role as both a biomarker and potential therapeutic target in preterm neonates, particularly those without severe hypoxia. Additionally, the HIF-1α pathway appears sensitive to CNS immaturity and warrants further investigation as a target for interventions aimed at mitigating neurological damage and improving outcomes in vulnerable newborns [38,39].

### 3.2. The Role of BDNF in Plasticity and Survival

BDNF plays a critical role in the adaptive response of the nervous system to perinatal insults, enhancing neuronal resistance to hypoxic–ischemic injury—especially in late gestation, when the brain undergoes rapid development [28,29]. Elevated BDNF expression may reflect a neuroprotective and pro-survival mechanism, promoting neurogenesis and facilitating brain repair [40]. Prematurity, however, is a significant risk factor for infant mortality, directly impacting the CNS due to the vulnerability of the germinal matrix [26]—a region rich in neuronal and glial precursors that is highly susceptible to hypoxic–ischemic injury owing to its fragile vascular structure [14,20]. Observational studies associate reduced BDNF levels with poorer outcomes in preterm neonates, including higher risks of retinopathy, lower survival rates, and neurodevelopmental delays [41,42,43], suggesting BDNF as both a mediator of perinatal adaptation and a prognostic marker in hypoxic–ischemic contexts [29].

Our study identified a positive correlation between survival time and BDNF expression (*p* = 0.012) in neonates without neuronal injury, indicating that longer survival is associated with higher BDNF levels. Further analysis demonstrated that an 11.82% increase in BDNF expression corresponded to an additional hour of survival (*p* < 0.001). These findings support the hypothesis that BDNF contributes not only to neural development but also serves as a dynamic prognostic indicator of survival in preterm neonates. Importantly, this association was more evident in the absence of overt neuronal injury, suggesting that preserved cerebral tissue may retain a greater capacity to mount BDNF-mediated neuroprotective responses, thereby promoting functional stability and prolonged survival [41,42].

The observation of higher BDNF levels in neonates without neuronal injury reinforces its potential role as a biomarker of neuronal integrity and clinical progression in preterm infants, particularly given the current lack of reliable biochemical markers for early postnatal brain injury assessment. In contrast, reduced BDNF expression in neonates with documented neuronal injury may reflect the metabolic and cellular stress experienced by damaged, yet still viable, neural tissue. Under such conditions, ongoing cellular stress and impaired intracellular signaling may limit the synthesis and availability of neurotrophic factors, including BDNF, a finding that is consistent with previous reports in hypoxic–ischemic brain injury [27,28,33].

Notably, these data suggest that BDNF expression may retain prognostic relevance in both asphyxiated and non-asphyxiated preterm neonates; however, the magnitude of its expression appears to be modulated primarily by the presence or absence of established neuronal injury rather than by asphyxia alone. This interpretation aligns with the concept that tissue integrity and metabolic viability are key determinants of neurotrophin production in the developing brain.

While a causal relationship between BDNF expression and neonatal outcomes cannot be inferred from this observational study, elevated BDNF levels appear to represent a favorable adaptive response associated with improved survival. Baseline BDNF expression may also reflect normal cerebral metabolic activity in the absence of significant hypoxic–ischemic damage [33]. Furthermore, the temporal dynamics of hypoxic–ischemic insults may influence the activation of upstream signaling pathways involved in BDNF regulation, such that acute or rapidly fatal insults may not allow sufficient time for full neurotrophic upregulation to occur. Further studies are therefore required to establish temporal and quantitative reference ranges for BDNF expression following hypoxic–ischemic injury.

If confirmed that BDNF elevation in preterm neonates contributes to improved survival, future research should explore its therapeutic potential to enhance endogenous neuroprotective mechanisms. Additionally, BDNF expression may serve as a valuable biomarker for prognostic assessment and for monitoring the effectiveness of neuroprotective interventions [29]. A deeper understanding of the regulatory pathways underlying BDNF expression may ultimately support the development of targeted strategies to improve neurological outcomes in vulnerable preterm populations [26,28].

At the molecular level, BDNF expression is regulated by upstream stress-responsive signaling pathways that integrate metabolic demand, tissue integrity, and temporal characteristics of hypoxic–ischemic insults. Experimental and translational evidence suggests that hypoxia-induced activation of HIF-1α may indirectly influence BDNF availability through intermediary signaling cascades involved in angiogenesis and cellular survival. Importantly, the activation of these pathways appears to be time-dependent, such that acute or rapidly evolving hypoxic–ischemic events may not allow sufficient progression of upstream signaling required for sustained BDNF upregulation. This temporal constraint may help explain the reduced BDNF expression observed in neonates with established neuronal injury, despite evidence of hypoxic stress. These mechanistic interactions are further explored in Section 3.4.

Taken together, these findings suggest that BDNF expression may reflect an adaptive neuroprotective response within the germinal matrix microenvironment, potentially representing an early molecular stage in the spectrum of hypoxic–ischemic stress preceding overt structural injury.

### 3.3. HIF-1α in Neonatal Hypoxia/Asphyxia

The neonatal period, particularly in preterm infants, is characterized by heightened vulnerability to hypoxic–ischemic injury, largely due to immature cerebral autoregulation. Consistent with current literature, our findings suggest that HIF-1α upregulation in response to hypoxia represents more than a marker of oxygen deprivation, functioning as an active adaptive mechanism to maintain cellular viability under metabolic stress [31]. This interpretation is supported by the statistically significant positive correlation between HIF-1α expression and survival in extremely preterm neonates (ρ = 0.492; *p* = 0.024), suggesting that hypoxia-driven angiogenic and metabolic responses may partially counteract systemic hypoxemia by optimizing tissue perfusion within the developing central nervous system [44].

Crucial to this discussion is the distinction between asphyxia—a global deprivation of oxygen and nutrients—and hypoxia, which often manifests as localized tissue oxygen deficiency preceding systemic compromise [10]. While HIF-1α activation reflects a generalized protective response, its biological efficacy is modulated by the duration of the insult, the tissue’s metabolic reserve, and gestational age [44].

Notably, while extremely preterm neonates showed a statistically significant association between HIF-1α expression and survival, late preterm neonates with neuronal injury exhibited a strong correlation that approached, but did not reach, conventional statistical significance (ρ = 0.949; *p* = 0.051). Although this association did not meet the predefined threshold for statistical significance, the magnitude of the correlation suggests a highly coordinated biological response that warrants cautious interpretation. This divergence between gestational groups likely reflects differences in vascular maturity and metabolic demand, indicating that HIF-1α activity may transition from a developmental role toward a survival-oriented response during periods of active neuronal differentiation and migration.

In term neonates, baseline HIF-1α expression is generally sufficient for cerebral development, with mature compensatory mechanisms reducing the reliance on HIF-mediated pathways except for during acute hypoxic stress [36]. However, the germinal matrix does not undergo uniform regression at term; rather, its involution is gradual and variable. Residual germinal matrix tissue in some term neonates may therefore remain susceptible to hypoxic–ischemic injury and hemorrhage, potentially contributing to periventricular leukomalacia even in more mature infants [45].

Regardless of gestational age, our data reinforce HIF-1α as a central mediator of neuroprotective responses, orchestrating angiogenesis (via VEGF), cellular survival pathways (including AKT/mTOR signaling), and neurotrophic support (including BDNF) within a tightly regulated metabolic network [25,26,27,28].

### 3.4. Interplay Between BDNF and HIF-1α

**Figure 5:**
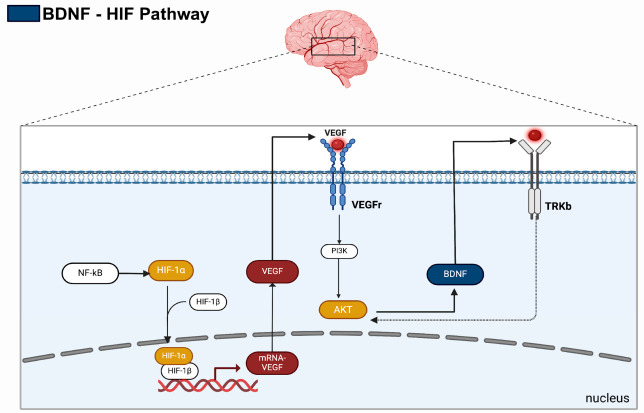
Hypothetical molecular model developed from the available literature and interpreted in the context of the findings of the present study. The model illustrates potential interactions between HIF-1α and BDNF signaling pathways in the germinal matrix during hypoxic stress. Under hypoxic conditions, NF-κB may contribute to HIF-1α stabilization, allowing its dimerization with HIF-1β and the transcriptional regulation of angiogenic and pro-survival genes, including *VEGF*. VEGF signaling through its receptor may activate downstream pathways such as PI3K/AKT, which are involved in cellular survival and plasticity. BDNF signaling via TrkB may also converge on PI3K/AKT activation, supporting a proposed integrated neuroprotective framework.

Building upon the temporal and molecular considerations discussed in the preceding sections, the interaction between HIF-1α and BDNF appears to be mediated by stress-responsive signaling pathways that integrate hypoxic burden, tissue integrity, and developmental stage. Rather than representing parallel or independent responses, HIF-1α and BDNF are increasingly recognized as components of a coordinated adaptive network, in which hypoxia-induced HIF-1α activation may indirectly modulate neurotrophic support through intermediary pathways involved in angiogenesis and cellular survival. Importantly, the activation and progression of these pathways are highly time-dependent, such that acute or rapidly evolving hypoxic–ischemic insults may elicit robust HIF-1α responses without sufficient temporal progression to sustain downstream neurotrophic upregulation (Figure 5).

Our data and Ghirelli et al. [34] identify PI3K/AKT, NF-κB, and VEGF (via VEGFR-1) as central to adaptive responses in preterms, coordinating hypoxic compensation and CNS maturation. Elevated AKT-3 in extremely immature neonates may indicate robust survival signaling, as BDNF-TrkB converges on PI3K/AKT to inhibit apoptosis and maintain neuronal integrity. AKT activation can upregulate *CREB*, increasing *BDNF* expression—suggesting AKT bridges HIF-1α (hypoxia-activated) and BDNF (CREB-mediated) pathways, forming a neuroadaptive cycle [34,35,36].

The study by Ghirelli et al. analyzed the same germinal matrix sample cohort used in the present investigation, focusing on the NF-κB/parkin/VEGFR-1 pathway. Consequently, the integration of these findings with the current analysis of HIF-1α and BDNF provides a more comprehensive view of the molecular mechanisms involved in hypoxic–ischemic injury within the germinal matrix [34].

NF-κB was upregulated in extremely immature neonates and those surviving >72 h, implicating its adaptive role in hypoxia. Despite its inflammatory associations, controlled NF-κB activity may stabilize HIF-1α and enhance survival signaling [34,37].

Higher VEGFR-1 in non-asphyxiated neonates supports VEGF’s role (by HIF-1α) in vascular homeostasis and neuronal survival [34,39]. These findings align with a model where HIF-1α activates *VEGF*, BDNF stimulates AKT, and NF-κB fine-tunes the response. Synergistic, regulated activation promotes angiogenesis, synaptic plasticity, and perfusion in vulnerable brain regions, mitigating injury [32,33]. However, persistent NF-κB hyperactivation may exacerbate inflammation and neurological deficits [37].

In summary, our research, along with the findings of Ghirelli et al. [34], substantiates that PI3K/AKT, NF-κB, and VEGF serve as crucial components within a neuroprotective network for extremely preterm neonates. Together with BDNF and HIF-1α, these mediators constitute an integrated system which, when equilibrated, enhances survival and mitigates brain injury in cases of extreme prematurity [31,35,37]. Therefore, interventions aimed at modulating HIF-1α, AKT, and VEGF, in conjunction with BDNF monitoring, may present promising neuroprotective strategies for at-risk preterm newborns.

## 4. Materials and Methods

### 4.1. Ethics Committee

This study was conducted in accordance with the Declaration of Helsinki and was reviewed and approved by the Research Ethics Committee (CEP) of the Pontifical Catholic University of Paraná (PUCPR), under protocol number 2533.140/2011-06. All procedures complied with the relevant institutional and national guidelines regarding the use of human autopsy tissues (Resolution CNS 466/12). Informed consent was obtained from the legal guardians of all neonates included in the study.

### 4.2. Samples

Fragments of the germinal matrix were obtained from the subependymal periventricular region corresponding to the ganglionic eminence, anatomically located adjacent to the head of the caudate nucleus. The samples were retrieved from the paraffin-embedded tissue bank of autopsies of neonates who died within the first 28 days of life (neonatal period) at the Hospital de Clínicas of the Universidade Federal do Paraná (Curitiba, Brazil) between 1991 and 2007.

A total of 118 CNS germinal matrix fragments were analyzed. Samples were categorized into three major groups based on gestational age: extremely preterm (<31 weeks; *n* = 56), late preterm (31–36 weeks; *n* = 36), and term neonates (≥37 weeks; *n* = 26). Perinatal asphyxia was operationally defined based on the 5 min Apgar score; neonates with scores ≤ 3 were classified as having Apgar-defined perinatal asphyxia, while scores > 3 were considered non-asphyxiated. Given the retrospective and autopsy-based nature of this cohort, the 5 min Apgar score was used as an available clinical proxy for severe perinatal compromise. Umbilical cord blood pH data were also available and were used as complementary biochemical information to support the clinical interpretation of metabolic acidemia and hypoxic–ischemic compromise (Supplementary Files).

**Figure 6:**
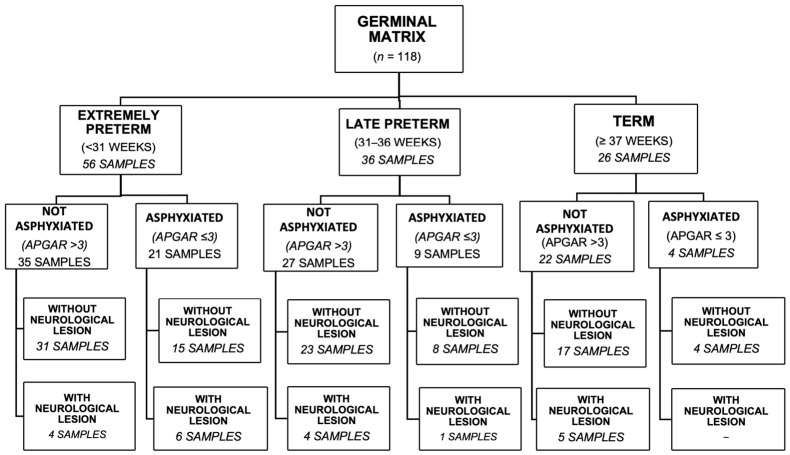
Flowchart of sample distribution across groups.

The presence of neurological injury was determined through macroscopic and microscopic examination of autopsy specimens. Figure 6 illustrates the sample distribution according to these criteria. All samples corresponded exclusively to the germinal matrix; no comparative analysis with hippocampal or cortical regions was performed.

### 4.3. Histological Analysis

The histopathological patterns in the samples were reviewed using hematoxylin and eosin (H&E) staining. The reagents utilized included Harris Hematoxylin (NewProv, Cat. PA203, Pinhais, Brazil) and Eosin (BIOTEC Reagentes Analíticos, Cat. 4371, Curitiba, Brazil). This analysis confirmed the integrity of the germinal matrix and identified specific histopathological alterations associated with hypoxic–ischemic insults.

Germinal matrix hemorrhage (GMH) was identified through macroscopic and histopathological criteria, including the presence of erythrocyte extravasation within the germinal matrix parenchyma and associated vascular disruption. Although hemorrhagic lesions differ morphologically from purely neuronal hypoxic–ischemic injury, GMH is widely recognized as a frequent structural consequence of hypoxic–ischemic stress in the immature germinal matrix. Therefore, these cases were included within the broader pathological spectrum of hypoxic–ischemic injury affecting the germinal matrix microenvironment.

### 4.4. Immunohistochemical Analysis

Immunohistochemistry was performed on tissue microarray (TMA) blocks. Representative areas of the germinal matrix were previously identified and marked on the H&E-stained slides. Cylindrical cores, approximately 0.3 cm in diameter, were extracted from the donor blocks and arranged into recipient TMA blocks.

Biomarker detection was performed using specific protocols for each molecule. For BDNF detection, sections were incubated with an anti-BDNF rabbit polyclonal primary antibody (Cat. No. E-AB-18244; Elabscience Bionovation Inc., Houston, TX, USA), diluted 1:200; For HIF-1α detection, sections were incubated with an anti-HIF-1α rabbit monoclonal primary antibody (clone EP118; Bio SB Inc., Santa Barbara, CA, USA), diluted 1:200. Incubations were conducted overnight in a humid chamber at 2–8 °C. Detection was carried out using the Mouse/Rabbit PolyDetector HRP/DAB kit (Bio SB Inc., Santa Barbara, CA, USA) for 20 min at room temperature. Signal development was achieved using 2,3-diaminobenzidine and hydrogen peroxide substrate, followed by Harris hematoxylin counterstaining. Results were validated using a known positive control sample exhibiting immunoreactivity for the respective antibodies.

### 4.5. Biomarker Analysis

Immunostained slides for BDNF and HIF-1α were scanned using the Axio Scan.Z1 system (Zen 2.6., Zeiss, Jena, Germany). Digital images were acquired, and 20 high-magnification fields (40× objective) were selected using ZEN Blue Edition software (Carl Zeiss Microscopy GmbH, Jena, Germany; available at: https://www.zeiss.com/microscopy/en/products/software/zeiss-zen.html, accessed on 4 June 2026). Image analysis was conducted blindly using randomly selected regions.

Immunopositivity areas were measured with Image Pro-Plus software, version 4.5 (Media Cybernetics, Rockville, MD, USA), using a semi-automated color segmentation method to isolate and quantify positive staining. The measured area (µm^2^) was divided by the total field area and converted to a percentage. Mean values from 20 fields per case were calculated and tabulated in Excel for statistical analysis.

### 4.6. Statistical Analysis

Data normality was assessed using the Shapiro–Wilk test. Given the non-parametric distribution of the data, comparisons between three or more groups were performed using the Kruskal–Wallis test, with results reported as medians, interquartile ranges (IQR), and minimum/maximum values. Spearman’s correlation coefficient was employed to evaluate the relationship between two variables. For BDNF-specific analysis, pairwise comparisons were conducted using the Bonferroni post hoc test.

Statistical significance was defined as a *p*-value ≤ 0.05. Primary immunopositivity data were analyzed using JMP Pro^®^ 14.0.0 software (SAS Institute, Cary, NC, USA). Additionally, a multiple regression model for BDNF was validated through residual hypothesis testing and the Breusch–Pagan test for homoscedasticity. In this specific model, BDNF expression (%) served as the dependent variable, while survival time (hours) was the independent variable. These advanced statistical analyses were performed using RStudio software (Posit PBC, Boston, MA, USA; available at: https://posit.co/products/open-source/rstudio/, accessed on 4 June 2026).

Given the retrospective nature of the cohort and the limited sample size of some stratified subgroups, subgroup analyses were considered exploratory. Therefore, correlation coefficients derived from small subgroups were interpreted with caution and regarded as hypothesis-generating rather than confirmatory.

### 4.7. AI Tools

During the preparation of this study, the authors utilized ChatGPT (OpenAI, GPT-4.1, 3 May 2025 Version) for academic translation into English and verification of reliability, and Grammarly (version 15.7.0) for grammar and style checks. The authors reviewed and edited the output and take full responsibility for the content of this study publication.

These AI tools do not meet authorship criteria and are not attributed as authors. The authors retain full responsibility for the content, originality, validity, and scientific integrity of the manuscript, including all sections developed with AI assistance.

## 5. Study Limitations

Some limitations should be considered when interpreting the findings of the present study. The assessment of tissue expression in formalin-fixed, paraffin-embedded (FFPE) postmortem specimens provides a single-point histopathological snapshot of the biological state of the germinal matrix at the time of death. Consequently, the observed immunohistochemical patterns reflect the molecular profile present at tissue collection and do not permit direct evaluation of the temporal dynamics underlying hypoxic–ischemic brain injury. This limitation is particularly relevant when interpreting the expression patterns of stress-responsive and neurotrophic markers, such as HIF-1α and BDNF, whose regulation is known to be time-dependent and influenced by the duration and severity of the insult. Therefore, HIF-1α and BDNF immunopositivity should not be interpreted as direct evidence of dynamic molecular changes occurring throughout the progression of hypoxic–ischemic injury.

To partially address this limitation, we employed statistical approaches incorporating survival time, including regression modeling, as a surrogate parameter to approximate temporal behavior. Although this strategy does not replace true longitudinal analysis, it provides an indirect framework for exploring associations between molecular expression and postnatal survival, allowing inference regarding potential temporal trends within the constraints imposed by postmortem pathology. Future prospective and experimental studies will be required to validate these relationships and to further characterize the temporal dynamics of HIF-1α and BDNF regulation following hypoxic–ischemic insults.

Another limitation relates to the operational definition of perinatal asphyxia, which was based primarily on the 5 min Apgar score. Although a low 5 min Apgar score is clinically relevant and reflects severe neonatal compromise, it does not fully encompass contemporary diagnostic criteria for perinatal asphyxia when considered in isolation. However, umbilical cord blood pH data were available and provided complementary biochemical evidence of metabolic acidemia. In addition, neonates classified as asphyxiated based on a 5 min Apgar score ≤ 3 exhibited neuropathological findings consistent with hypoxic–ischemic injury at autopsy, supporting the biological plausibility of case classification. Nevertheless, the asphyxia-related analyses should be interpreted within the context of an operational definition supported by clinical, biochemical, and neuropathological findings rather than a comprehensive contemporary diagnostic assessment including standardized neurological staging, neuroimaging, electroencephalography, and multiorgan dysfunction criteria.

Finally, the relatively small sample size in some subgroup analyses may have limited statistical power and reduced the precision of the correlation estimates. Therefore, the associations observed in these analyses should be interpreted with caution and considered exploratory until confirmed in larger cohorts. Such limitations are not uncommon in studies involving the human germinal matrix, a transient neurodevelopmental structure with inherently restricted availability for research and often represented by small tissue specimens. As a result, sample size constraints remain an important methodological consideration in neuropathological investigations of this region.

## 6. Conclusions

The findings of this study demonstrate that the immunopositivity of BDNF and HIF- 1α in the germinal matrix is associated with distinct clinical and neuropathological features of neonatal hypoxic–ischemic injury, in a manner that is strongly influenced by gestational maturity and tissue integrity. Rather than reflecting uniform responses, the expression patterns of these markers appear to vary according to the presence of neuronal injury, survival time, and the developmental stage of the brain.

Increased BDNF expression was associated with prolonged postnatal survival in preterm neonates without overt neuronal injury, reinforcing the need for future translational and in vivo studies to determine whether BDNF may serve as a prognostic biomarker of tissue viability and adaptive neuroprotection. In contrast, reduced BDNF expression in cases with established neuronal injury may reflect ongoing cellular stress and impaired neurotrophic capacity within damaged, yet viable, neural tissue.

HIF-1α immunopositivity was associated with survival and neuronal injury in a gestational age-dependent manner, consistent with its role as an immediate sensor of hypoxic stress and a regulator of adaptive metabolic and angiogenic responses. These findings suggest that HIF-1α activity may reflect both developmental and injury-related processes within the germinal matrix, particularly under conditions of hypoxic–ischemic challenge.

Collectively, the results underscore the relevance of the germinal matrix as a metabolically active and vulnerable structure in both preterm and selected term neonates, where neuroprotective and stress-response pathways may be differentially engaged. Although the present study is limited by its postmortem and cross-sectional design, the integration of molecular, clinical, and survival data provides a biologically plausible framework for understanding maturity- and time-dependent responses to hypoxic–ischemic injury.

Future studies employing longitudinal, experimental, and translational approaches will be essential to validate the temporal dynamics of BDNF and HIF-1α regulation and to explore their potential utility as prognostic markers or therapeutic targets in neonatal hypoxic–ischemic conditions.

## Figures and Tables

**Table 1:** Data from the samples used with their respective clinical characteristics.

Data	Category	Number of Patients (%)	Mean ± SD
Sex	Male	(66) 55.9%	-
	Female	(52) 44.1%	
Newborn Weight (g)	<1000	(34) 28.8%	171 ± 975
	1000–1499	(29) 24.6%	
	≥1500	(55) 46.6%	
Gestational Age (weeks)	≤30 weeks and 6 days	(56) 47.5%	26.73 ± 2.13
	31 to 36 weeks and 6 days	(36) 30.5%	33.63 ± 1.49
	≥37	(26) 22.0%	38.88 ± 1.31
Asphyxia (Apgar score ≤ 3 at 5 min)	No	(84) 71.2%	5.6 ± 2.8
	Yes	(34) 28.8%	
Survival (hours)	Up to 24 h	(38) 32.2%	4.4 ± 8
	Between 25 and 72 h	(48) 40.7%	
	More than 72 h	(32) 27.1%	
Anatomopathological Findings	Periventricular and intraventricular hemorrhage, cerebral edema and congestion, hypoxic type neuronal changes, leptomeningeal congestion and germinal matrix hemorrhage.		

**Table 2:** Simple linear regression analysis between BDNF immunopositivity and postnatal survival time. Model statistics: R^2^ = 0.091; Adjusted R^2^ = 0.079; F (1.75) = 7.5336; *p* = 0.008.

Variable	β Coefficient (Estimate)	Standard Error	t-Value	*p*-Value
Intercept	75.455	21.097	3.577	<0.001
BDNF (%)	11.823	4.307	2.745	0.008

## Data Availability

The data supporting the findings of this study are available from the corresponding author upon reasonable request.

## Supplementary Materials

The following supporting information can be downloaded at: https://www.mdpi.com/article/10.3390/ijms27115125/s1.
