# Supplementary material for: The Involvement of HIF-1α and BDNF in Neonatal Hypoxic–Ischemic Insult to the Cerebral Germinal Matrix
Source: Int J Mol Sci. 2026 Jun 5;27(11):5125. doi: 10.3390/ijms27115125 (PMC13257392; doi:10.3390/ijms27115125)
Supplement: Supplementary file 1 [file ijms-27-05125-s001.zip › Ethic approval.pdf]

HOSPITAL DE CLÍNICAS
UNIVERSIDADE FEDERAL DO PARANÁ

CEP/HC/UFPR

Curitiba, 04 de julho de 2011.

Ilmo (a) Sr. (a)
Francisco Cesar Pabis
Hospital de Clinicas da UFPR
Curitiba - PR

Prezado Pesquisador:

Comunicamos que o Projeto de Pesquisa intitulado: **"AVALIAÇÃO IMUNOISTOQUIMICA DE BIOMARCADORES DE CRESCIMENTO CELULAR EM AMOSTRAS DE MIOCARDIO DE PREMATUROS HIPOXEMIADOS"**, foi analisado e aprovado pelo Comitê de Ética em Pesquisa em Seres Humanos, em reunião realizada no dia 28 de junho de 2011.

O referido projeto atende aos aspectos das Resoluções CNS 196/96, e complementares, sobre Diretrizes e Normas Regulamentadoras de Pesquisa Envolvendo Seres Humanos do Ministério da Saúde.

CAAE: 0139.0.208.000-11
Registro CEP: 2534.141/2011-06

Conforme a Resolução 196/96, solicitamos que sejam apresentados a este CEP, relatórios sobre o andamento da pesquisa, bem como informações relativas às modificações do protocolo, cancelamento, encerramento e destino dos conhecimentos obtidos.

Data para entrega do primeiro relatório: janeiro de 2012.

Atenciosamente,

Renato Tambara Filho
Coordenador do Comitê de Ética em Pesquisa
em Seres Humanos do Hospital de Clinicas/UFPR
