# Supplementary material for: The Involvement of HIF-1α and BDNF in Neonatal Hypoxic–Ischemic Insult to the Cerebral Germinal Matrix
Source: Int J Mol Sci. 2026 Jun 5;27(11):5125. doi: 10.3390/ijms27115125 (PMC13257392; doi:10.3390/ijms27115125)
Supplement: Supplementary file 1 [file ijms-27-05125-s001.zip › Informed Consent Form.pdf]

HOSPITAL DE CLÍNICAS
UNIVERSIDADE FEDERAL DO PARANÁ
UFPR
EBSERH
HOSPITAIS UNIVERSITÁRIOS FEDERAIS
Ministério da Educação
GOVERNO FEDERAL BRASIL PÁTRIA EDUCADORA

# TERMO DE CONSENTIMENTO LIVRE E ESCLARECIDO PARA REALIZAÇÃO DE NECROPSIA

Por este instrumento eu, ____________________________________________(nome completo), __________________________ (grau de parentesco com o paciente), RG ____________________, autorizo a necropsia (exame do corpo do paciente após o seu óbito) para fins de determinação da causa da morte, de doenças primárias, doenças não diagnosticadas e correlacionar diagnósticos prévios de____________________________________________________________
______________________________________________________________
______________________________________________________________
______________________________________________________________
______________________________________________________________

Nome do paciente: ______________________________RG-HC Nº.: __________

Data do óbito: ____/____/_________ Hora do óbito: ______________________

Unidade do CHC onde ocorreu o óbito: ______________________________

Declaro estar ciente que:

a) O procedimento implicará na retirada de todos os órgãos do corpo para exames complementares e que as amostras não utilizadas, serão posteriormente incineradas.

**b) Por determinação legal, a realização da necropsia iniciará após 6 horas a partir do horário do óbito e, ainda serão necessárias mais algumas horas, para realização do procedimento, conforme cada caso, até poder ser feita a liberação do corpo para família;**

c) O prazo para o resultado final do exame de necropsia é de no mínimo 90 dias;

c) Esta necropsia **não tem caráter pericial**, não sendo indicada em casos de morte por causas não naturais como homicídio, suicídio, acidente ou morte suspeita, não importando o tempo entre o evento violento e a morte;

d) Também autorizo a utilização das amostras biológicas provenientes do exame de necropsia para ensino e pesquisa, desde que respeitadas as normas vigentes no país, para este uso;

Sendo assim, expresso meu pleno, livre e esclarecido consentimento, para a realização da necropsia no Complexo Hospital de Clinicas da UFPR.

Ass. do parente e/ou responsável: ______________________________

Ass., nome e CRM do médico solicitante: ______________________________

Curitiba, _____ de _____________de ______.

Este documento deve ser preenchido em uma única via de forma completa, ser assinado por todos e anexado ao prontuário do paciente.
