# Supplementary material for: The Involvement of HIF-1α and BDNF in Neonatal Hypoxic–Ischemic Insult to the Cerebral Germinal Matrix
Source: Int J Mol Sci. 2026 Jun 5;27(11):5125. doi: 10.3390/ijms27115125 (PMC13257392; doi:10.3390/ijms27115125)
Supplement: Supplementary file 1 [file ijms-27-05125-s001.zip › Supplementary Figure S1.pdf]

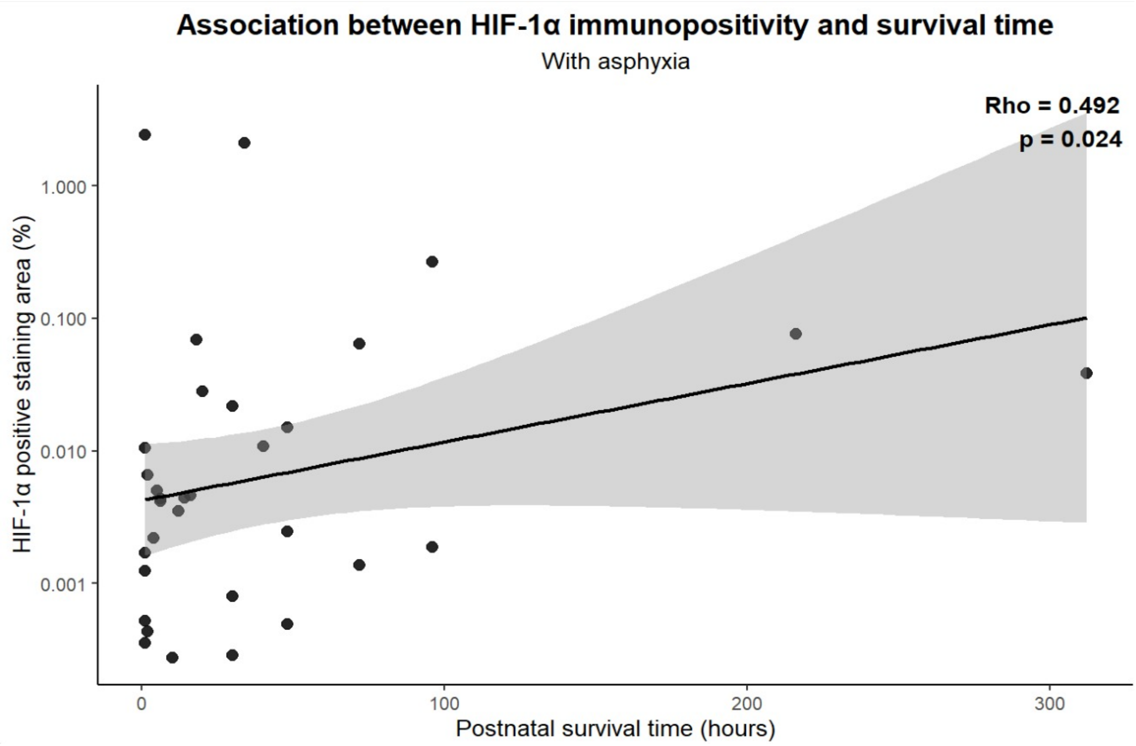

Supplementary Figure S1. **Scatterplot showing the association between HIF-1α immunopositivity and postnatal survival time in asphyxiated neonates.** Each point represents an individual neonate. HIF-1α positive staining area (%) is displayed on a logarithmic scale. The solid line represents the linear trend and the shaded area indicates the 95% confidence interval. A significant positive correlation was observed between HIF-1α immunopositivity and postnatal survival time (Spearman's Rho = 0.492, p = 0.024).
