# Supplementary material for: The Involvement of HIF-1α and BDNF in Neonatal Hypoxic–Ischemic Insult to the Cerebral Germinal Matrix
Source: Int J Mol Sci. 2026 Jun 5;27(11):5125. doi: 10.3390/ijms27115125 (PMC13257392; doi:10.3390/ijms27115125)
Supplement: Supplementary file 1 [file ijms-27-05125-s001.zip › Supplementary Table S1.pdf]

Supplementary Table S1. Conceptual relationships underlying the chord diagram

Source	Target	Weight	Brief justification
BDNF	Synaptic Plasticity	10	BDNF is a key factor for synaptic remodeling and strengthening
BDNF	Neuroprotection	9	BDNF reduces neuronal damage and promotes neuronal survival
BDNF	Survival	7	Findings suggest a correlation between BDNF levels and improved prognosis
HIF-1	Hypoxia	10	Classical transcription factor involved in the hypoxic response
HIF-1	Asphyxia	8	In asphyxiated neonates, HIF-1 may be upregulated as an adaptive response
HIF-1	Neuronal Injury	6	HIF-1 regulates genes that may contribute to neuroprotection, depending on the context
Late Preterm (LP)	HIF-1	7	Stronger correlations observed in asphyxiated LP neonates, suggesting a role of HIF-1 in survival
Extremely Preterm (EP)	BDNF	8	Elevated BDNF levels were observed in non-asphyxiated EP neonates
Extremely Preterm (EP)	Neuronal Injury	5	Evidence of correlation in subgroups with neuronal injury, although borderline
Synaptic Plasticity	Neuroprotection	8	Synaptic plasticity supports recovery and synaptic reorganization
Neuronal Injury	Survival	4	Neuronal injury may compromise survival, with variability across subgroups
Asphyxia	Hypoxia	9	Neonatal asphyxia characterizes a state of systemic or local hypoxia

Supplementary Table S1. Conceptual relationships underlying the chord diagram. *Weights reflect the relative strength and consistency of statistically significant associations observed in the study, integrated with their biological relevance. These weights are intended for conceptual synthesis and visualization purposes and do not correspond to formal statistical coefficients.*
